# The impact of the COVID-19 pandemic on social determinants of health, mental health, and substance use among key populations affected by sexually transmitted and blood-borne infections in Canada

**DOI:** 10.17269/s41997-024-00888-4

**Published:** 2024-04-30

**Authors:** Herak Apelian, Josephine Aho, Elsie Wong, Joseph Cox

**Affiliations:** Sexually Transmitted and Blood-Borne Infections Surveillance Division, Public Health Agency of Canada, Ottawa, ON Canada

**Keywords:** Social determinants of health, Mental health, Substance use, Key populations, Déterminants sociaux de la santé, santé mentale, consommation de substances, populations clés

## Abstract

**Objectives:**

We assessed the impact of the COVID-19 pandemic on social determinants of health, mental health, substance use, and access to mental health and harm reduction services among key populations disproportionately impacted by sexually transmitted and blood-borne infections (STBBI).

**Methods:**

Online surveys (2021‒2022) were conducted in Canada among people who use drugs or alcohol (PWUD); African, Caribbean, and Black people (ACB); and First Nations, Inuit, and Métis peoples (FNIM). Descriptive analyses were conducted on social determinants of health, substance use, and access to services, stratified by changes in mental health status since the start of the pandemic.

**Results:**

A total of 3773 participants (1034 PWUD, 1556 ACB, and 1183 FNIM) completed the surveys, with 45.6% reporting a major/moderate impact of the pandemic on their ability to pay bills and 53% experiencing food insecurity since the start of the pandemic. Half (49.4%) of participants reported deteriorating mental health. A higher increase in substance use and related behaviours was seen in those with worsening mental health. Among those using substances, two thirds (69.4%) of those with worsening mental health reported increasing their use of substances alone, compared to 46.9% of those with better/similar mental health. Access to mental health and harm reduction services was low.

**Conclusion:**

These intersecting health issues are among the risk factors for STBBI acquisition and act as barriers to care. Equitable interventions and policies addressing downstream and upstream determinants of health, with meaningful and sustainable leadership from key populations, may improve their health and well-being, to lower STBBI impact and improve future pandemic responses.

## Résumé

**Objectifs:**

Nous avons évalué l’impact de la pandémie de COVID-19 sur les déterminants sociaux de la santé, la santé mentale, la consommation de substances et l’accès aux services de santé mentale et de réduction des méfaits des populations clés qui sont affectées de manière disproportionnée par les infections transmissibles sexuellement et par le sang (ITSS).

**Méthodes:**

Des enquêtes en ligne (2021‒2022) ont été menées au Canada auprès de personnes utilisatrices de drogues ou d’alcool (PUD), de personnes africaines, caribéennes et noires (ACN) et des peuples des Premières Nations, des Inuits et des Métis (PNIM). Des analyses descriptives ont été effectuées sur les déterminants sociaux de la santé, la consommation de substances et l’accès aux services, stratifiés selon les changements dans l’état de santé mentale depuis le début de la pandémie.

**Résultats:**

Un total de 3 773 participants (1 034 PUD, 1 556 ACN et 1 183 PNIM) ont complété les enquêtes, dont 45,6 % ont rapporté un impact majeur/modéré de la pandémie sur leur capacité à payer leurs factures et 53 % ont vécu de l’insécurité alimentaire depuis le début de la pandémie. La moitié (49,4 %) des participants ont signalé une détérioration de leur santé mentale. Une augmentation plus élevée de la consommation de substances et des comportements liés a été observée chez ceux dont la santé mentale s’est détériorée. Parmi ceux qui consommaient des substances, deux tiers (69,4 %) de ceux dont la santé mentale s’était détériorée ont signalé une augmentation de leur consommation solitaire de substances, comparativement à 46,9 % de ceux dont la santé mentale était meilleure ou similaire. L’accès aux services de santé mentale et de réduction des méfaits était faible.

**Conclusion:**

Ces problèmes de santé croisés font partie des facteurs de risque d’acquisition des ITSS et constituent des obstacles aux soins. Des interventions et des politiques équitables, tenant compte des déterminants de la santé en amont et en aval, avec un leadership significatif et durable des populations clés, peuvent améliorer leur santé et leur bien-être, réduire l’impact des ITSS et améliorer les réponses aux futures pandémies.

## Introduction

Structural inequities such as racial discrimination and economic disparities produce systematic disadvantages and create an environment in which social determinants of health are impacted (Baciu et al., 2017; Public Health Agency of Canada (PHAC), 2023b). These determinants, like unemployment and food insecurity, can particularly affect key populations that are disproportionately impacted by sexually transmitted and blood-borne infections (STBBI) in Canada. These include people who use drugs or alcohol (PWUD); African, Caribbean, and Black people (ACB); and First Nations, Inuit, and Métis peoples (FNIM) (PHAC, 2024). FNIM peoples face inequities in health as a result of systemic and structural factors, such as colonization, loss of language and culture, and intergenerational trauma (Jongbloed et al., 2019). ACB people face systemic anti-Black racism that stems from historical slavery roots and extends to the education system and the criminal justice system (Collaborative Critical Research for Equity and Transformation in Health (CO-CREATH), 2022a; Department of Justice Canada, 2023; Ezezika et al., 2023). Unique challenges for PWUD include precarious living and stigma associated with and criminalization of substance use (Russell et al., 2021). Taken together, these inequities can have an effect on social determinants of health, mental health, and substance use and represent risk factors for infectious diseases, including STBBI, and act as barriers to care (Baciu et al., 2017; Jongbloed et al., 2019). As highlighted in the pan-Canadian STBBI Framework for Action, a focus on key populations is crucial to reduce the health impact of STBBI in Canada by 2030 and to achieve the World Health Organization 2030 global STBBI targets (PHAC, 2018). The Government of Canada’s recently published renewed 2024‒2030 STBBI Action Plan builds on this work and continues the focus on health equity and equity-deserving populations (PHAC, 2024).

The COVID-19 pandemic has had a negative impact on social determinants of health, such as food insecurity and domestic violence (PHAC, 2021), and on the mental health of Canadians. Findings from national surveys, conducted throughout different waves of the pandemic from 2020 to 2022, showed a significant increase in negative psychosocial outcomes relative to pre-COVID levels (Brotto et al., 2021), including elevated mental health concerns and substance use in regions across Canada (Canadian Centre on Substance Use and Addiction (CCSU), 2023). The interplay among social determinants of health, mental health, and substance use is well documented (Brotto et al., 2021; CCSU, 2023; Men et al., 2021; Mental Health Commission of Canada, 2024; Newfoundland and Labrador (NL) Health Services, 2023). For example, discrimination is generally associated with negative mental health outcomes (Baciu et al., 2017). There is also research suggesting that perceived danger and fear of violence can have an influence on stress and substance use (Baciu et al., 2017).

While these changes in social determinants of health and mental health were reported in the general population, the COVID-19 pandemic may have had a greater impact on psychosocial and health outcomes among key populations affected by STBBI in Canada. In this work, we describe the collective results of three surveys conducted among three key population groups: PWUD, ACB, and FNIM. These surveys aimed to explore the impacts of COVID-19 on social determinants of health, mental health, substance use, and access to mental health and harm reduction services. We present indicators related to social determinants of health, as well as indicators related to substance use and access to services stratified by changes in mental health status since the start of the COVID-19 pandemic.

## Methods

### Study design

Three anonymous, self-administered, online surveys were conducted by the Public Health Agency of Canada (PHAC) in 2021‒2022 through the Tracks Surveillance System. The survey design was inspired by the rapid assessment trendspotter methodology used in the European Monitoring Centre for Drug Addiction’s online survey on the impact of COVID-19 (European Monitoring Centre for Drugs and Drug Addiction, 2020).

To ensure community engagement in the planning, promotion, and recruitment of the surveys, PHAC collaborated with different partners for each survey. Working groups were formed and contributed to protocol and questionnaire design, promotion of the survey during data collection, and review of knowledge translation products. For the survey among PWUD, key stakeholders included the Canadian Association of People who Use Drugs (CAPUD). CAPUD established and led a national expert working group comprised of: (1) people with lived and/or had living experience of drug use, among whom some were individuals from the African, Caribbean, and Black; Indigenous; and 2SLGBTQIA + communities; (2) representatives from community-based organizations offering STBBI-related services, academic institutions, and advocacy groups; and (3) community-engaged researchers.

For the survey among ACB, key stakeholders included the University of Ottawa and Women’s Health in Women’s Hands, who assembled the national expert working group (NEWG). NEWG, an innovative community-based participatory approach, consisted of ACB researchers, service providers, and community members and leaders. NEWG referred to this survey as the African, Caribbean and Black Community PHAC-funded COVID-19 Impact (APCI) study. The group promoted the survey through various methods to ensure appropriate representation across diverse ACB sub-populations and led the dissemination of results through a report (CO-CREATH, 2022a) and a community event (CO-CREATH, 2022b).

The survey among FNIM was conducted in collaboration with the National Collaborating Centres for Indigenous Health (NCCIH) and Infectious Diseases (NCCID) which established a National Indigenous STBBI Advisory Committee. The committee comprises Indigenous experts in the STBBI and public health sector. Analyses of these data are underway, led by NCCIH and NCCID, with the support of the Advisory Committee.

### Participants and data collection

Eligible FNIM or ACB participants were aged 15 + (FNIM) or 18 + (ACB), lived in Canada, and self-identified as FNIM or ACB, respectively. Eligible PWUD participants were aged 18 + , lived in Canada, and reported any substance use including alcohol or cannabis 6 months prior to data collection. Survey links were distributed through (1) PHAC, (2) stakeholders, and (3) working groups detailed above. Data collection for the PWUD arm started first, from January to February 2021, followed by ACB, from May to July 2021, and FNIM, from July 2021 to January 2022. Given that these were three distinct surveys, participants could have potentially completed more than one survey if they self-identified as belonging to more than one key population (i.e. PWUD identifying as FNIM or ACB). However, this proportion was low (< 11%).

### Variables and statistical analysis

The three surveys were structured similarly with comparable content. There were minor differences in a few questions based on community needs and realities identified by community partners. Information was collected on sociodemographic characteristics, changes in social determinants of health (employment and financial security, food security, feelings of safety, domestic violence), mental health, substance use, and access to mental health, harm reduction, and STBBI-related services, since the start of the pandemic. Using pooled data, descriptive analyses were conducted to present indicators related to social determinants of health and selected indicators of substance use and access to services stratified by changes in mental health status. The latter is based on the following question: “Compared to before the COVID-19 pandemic started, how would you say your mental health is now?” Participants were stratified into two groups: those who answered, “much better now”, “somewhat better now”, or “about the same” and those who answered, “somewhat worse now” and “much worse now”. All analyses were conducted using R statistical software.

### Ethics approval

The survey protocol and questionnaire were approved by the Health Canada/PHAC Research Ethics Board (REB 2020-013P).

## Results

A convenience sample of 3773 participants (1034 PWUD, 1556 ACB, and 1183 FNIM [70.4% First Nations; 23.7% Métis; 4.5% Inuit; 1.4% other]) completed the respective surveys (Table 1). The majority of participants were from Ontario (39.7% of PWUD, 42.7% of ACB, and 25.8% of FNIM). The two provinces with the higher proportion of participants were British Columbia (14.1%) and Nova Scotia (9.4%) for PWUD; Quebec (12.7%) and British Columbia/Alberta (10.9%) for ACB; and British Columbia (24.2%) and Manitoba (13.4%) for FNIM. Most participants were cisgender women (61.2–80.9%).

**Table 1:** Sociodemographic characteristics of PWUD, ACB, and FNIM participants

Characteristic	PWUD (*n* = 1034)			ACB (*n* = 1556)			FNIM (*n* = 1183)		
	*n*	Total^a^	%	*n*	Total	%	*n*	Total	%
Province or territory where participant lives									
British Columbia	146	1032	14.1	169	1554	10.9	286	1183	24.2
Alberta	75	1032	7.3	170	1554	10.9	170	1183	14.4
Saskatchewan	39	1032	3.8	30	1554	1.9	86	1183	7.3
Manitoba	46	1032	4.5	19	1554	1.2	158	1183	13.4
Ontario	410	1032	39.7	664	1554	42.7	305	1183	25.8
Quebec	126	1032	12.2	197	1554	12.7	42	1183	3.6
New Brunswick	41	1032	4	62	1554	4	32	1183	2.7
Nova Scotia	97	1032	9.4	54	1554	3.5	33	1183	2.8
Prince Edward Island	7	1032	0.7	67	1554	4.3	4	1183	0.3
Newfoundland and Labrador	31	1032	3	81	1554	5.2	31	1183	2.6
Territories^b^	14	1032	1.4	39	1554	2.5	31	1183	2.5
None of the above^c^	0	1032	0	2	1554	0.1	5	1183	0.4
Age group									
Younger than 25 years	105	1034	10.2	178	1556	11.4	64	1183	5.4
25 to 39 years	449	1034	43.4	616	1556	39.6	279	1183	23.6
40 to 54 years	298	1034	28.8	519	1556	33.4	385	1183	32.5
55 years or older	182	1034	17.6	243	1556	15.6	455	1183	38.5
Gender identity^d^									
Cisgender female	622	1016	61.2	990	1496	66.2	927	1146	80.9
Cisgender male	331	1016	32.6	462	1496	30.9	142	1146	12.9
Transfeminine^e^	20	1016	2	11	1496	0.7	15	1146	1.3
Transmasculine^f^	43	1016	4.2	33	1496	2.2	62	1146	5.4
Citizenship status									
Canadian citizen, born in Canada	923	1032	89.4	358	1546	23.2	NA	NA	NA
Canadian citizen, not born in Canada	85	1032	8.2	630	1546	40.8	NA	NA	NA
Landed immigrant or permanent resident	17	1032	1.7	291	1546	18.8	NA	NA	NA
Convention refugee or temporary resident	7	1032	0.7	254	1546	16.4	NA	NA	NA
Not a Canadian citizen — other	0	1032	0	13	1546	0.8	NA	NA	NA
Education, highest level									
Less than or some high school	61	1024	6	67	1462	4.6	96	1146	8.4
Finished high school	80	1024	7.8	105	1462	7.2	221	1146	19.3
Some or completed college, CEGEP, vocational school, trade school, or apprenticeship training	279	1024	27.2	253	1462	17.3	645	1146	56.3
Some or completed university certificate or diploma, undergraduate university degree, or graduate or professional university degree	599	1024	58.5	1027	1462	70.2	160	1146	14.0
Other	5	1024	0.5	10	1462	0.7	24	1146	2.1

The sum of the percentages may not equal 100% due to rounding, unless stated otherwise

*STBBI*, sexually transmitted and blood-borne infection; -, indicates data was suppressed due to small cell counts

^a^Total represents total counts for the corresponding indicator excluding “don’t know”, “refused”, and “not stated” values, unless stated otherwise

^b^Includes Nunavut, Yukon, and Northwest Territories

^c^Respondents were eligible to participate if they reported that they were in Canada at the time of enrollment. While all respondents represented in these tables met all eligibility criteria, participants were provided the option to select “None of the above” as a valid answer to this question

^d^The Multidimensional Sex/Gender Measure was used to measure gender identity (Bauer et al., 2017)

^e^Transfeminine included those assigned male at birth who identified with either female or a non-binary gender

^f^Transmasculine included those assigned female at birth who identified with either male or a non-binary gender

### Social determinants of health

Regarding social determinants of health, more than half of all participants (53%) reported experiencing food insecurity while almost half of participants (45.6%) reported the pandemic had a “major or moderate” impact on their ability to pay bills (Table 2). Precarious housing across all three samples was 8.5%. Almost a quarter said they were feeling less safe at home. Experiencing any kind of abuse since the start of the COVID-19 pandemic ranged from 28.2% to 56.3%. Among these, 23.4–53.4% reported experiencing abuse more often since the start of the pandemic.

**Table 2:** Overview of social determinants of health among PWUD, ACB, and FNIM participants

Indicator	All participants		
	*n*	Total^a^	%
Housing status^b^			
Stable housing^c^	3405	3772	91.5
Precarious or inadequate housing^d^	367	3772	8.5
Financial security			
Change in work situation since the start of the COVID-19 pandemic: reduced hours and/or pay or had to stop working	1311	3592	36.5
Impact of the COVID-19 pandemic on ability to pay bills: major and moderate	1638	3589	45.6
Food security			
Experienced food insecurity since the start of the COVID-19 pandemic^e^	1801	3398	53.0
Feelings of safety and domestic violence			
Change in self-reported safety where participant lives since the start of the COVID-19 pandemic: less safe			
Among all participants	760	3350	22.7
Among those who felt very safe in the home in the year before the pandemic	355	2025	17.5
Among those who felt somewhat safe or did not feel safe in the home in the year before the pandemic	393	1245	31.6
Since the start of the COVID-19 pandemic:			
Experienced verbal abuse: any	1663	2955	56.3
Among participants who experienced verbal abuse Change in frequency of verbal abuse: more often	888	1663	53.4
Experienced physical abuse: any	854	2903	29.4
Among participants who experienced physical abuse Change in frequency of physical abuse: more often	262	854	30.7
Experienced sexual aggression: any	806	2863	28.2
Among participants who experienced sexual abuse Change in frequency of sexual aggression: more often	189	806	23.4
Experienced financial abuse: any	951	2881	33.0
Among participants who experienced financial abuse Change in frequency of financial abuse: more often	300	951	31.5
Mental health			
Change in mental health status since the start of the COVID-19 pandemic: somewhat or much worse now			
Among all participants	1864	3770	49.4
Among those with excellent, very good, or good mental health at the time of the survey	746	2267	32.9
Among those with fair or poor mental health at the time of the survey	1117	1502	74.4

^a^Total represents total counts for the corresponding indicators excluding “don’t know”, “refused”, and “not stated” values, unless stated otherwise, which is why the total counts differ by indicator

^b^This indicator measured the participant’s living situation since the start of the COVID-19 pandemic. Participants could report more than one type of living situation

^c^Participants were classified as living in stable housing if they were only living in their own apartment or house, or in a relative’s or friend’s place

^d^If a participant indicated living in any of the following precarious housing situations, they were classified as living in precarious or inadequate housing: living in multiple residences or couch surfing, a hotel or motel room, rooming or boarding house, shelter or hostel, transition or halfway house, psychiatric institution or drug treatment facility, public place, or correctional facility

^e^Participants were classified as experiencing food insecurity if they indicated “Often true” or “Sometimes true” to any of the following food insecurity situations since the start of the COVID-19 pandemic: you or other household members couldn’t afford to eat healthy and balanced meals; you ate less than you felt you should because there wasn’t enough money to buy food; others in your household ate less than you felt they should because there wasn’t enough money to buy food; you or other household members accessed food or meals, at no cost to you, from a community organization

### Mental health and substance use

Among all participants, 49.4% reported their mental health was “somewhat or much worse” since the start of the pandemic. Compared to participants who reported their mental health was “same or better” since the start of the pandemic, a higher proportion of participants with “somewhat or much worse” mental health reported increasing their substance use and risks associated with substance use (Table 3). For example, 71.4% of participants with worsening mental health reported using alcohol since the start of the pandemic; among them, 57.9% reported an increase in their use. In contrast, 55.1% of participants with “same or better” mental health reported using alcohol and 36.1% reported an increase in their use. Among other substance use behaviours, a higher proportion of participants with worsening mental health reported an increase in the number of different triggers for using substances (83% vs. 52.8% among participants with same/better mental health) and an increase in using substances alone (69.4% vs. 46.9% among participants with same/better mental health).

**Table 3:** Mental health, substance use, and access to services among PWUD, ACB, and FNIM participants

	Change in mental health status since the start of the pandemic (*n* = 3770)					
	Much better, somewhat better, about the same (*n* = 1906)			Somewhat or much worse now (*n* = 1864)		
	*n*	Total^a^	%	*n*	Total^a^	%
Substance use						
Drug used since the start of the COVID-19 pandemic						
Alcohol: used	850	1544	55.1	1082	1515	71.4
Among those who used alcohol						
Increase use of alcohol	307	850	36.1	627	1082	57.9
Cannabis: used	514	1546	33.2	791	1513	52.3
Among those who used cannabis						
Increase use of cannabis	261	514	50.8	550	791	69.5
Cocaine/crack: used	109	1552	7.0	159	1512	10.5
Among those who used cocaine/crack						
Increase use of cocaine/crack	42	109	38.5	84	159	52.8
Speed/meth: used	81	1554	5.2	136	1513	9.0
Among those who used speed/meth						
Increase use of speed/meth	28	81	34.6	78	136	57.4
Hallucinogens: used	159	1556	10.2	201	1509	13.3
Among those who used hallucinogens						
Increase use of hallucinogens	58	159	36.5	102	201	50.7
Ecstasy: used	100	1556	6.4	104	1507	6.9
Among those who used ecstasy						
Increase use of ecstasy	19	100	19.0	35	104	33.7
Heroin, fentanyl or other opioids: used	81	1563	5.2	104	1511	6.9
Among those who used heroin						
Increase use of heroin, fentanyl or other opioids	29	81	35.8	56	104	53.8
Substance use behaviours						
Among those who used substances						
Increases in substance consumption behaviours, since the start of the COVID-19 pandemic						
Had different triggers for using	153	290	52.8	512	617	83.0
Used alone	149	318	46.9	387	558	69.4
Used substances not usually consumed	82	191	42.9	232	376	61.7
Had withdrawal symptoms	73	176	41.5	202	343	58.9
Worried about overdosing	53	157	33.8	139	303	45.9
Shared used equipment such as needles or syringes, pipes, tourniquets, swabs, and cookers	22	108	20.4	53	198	26.8
Was unable to get the substances used	67	222	30.2	157	380	41.3
Access to mental health services						
Accessed or considered accessing mental health and wellness services since the start of the COVID-19 pandemic	777	1877	41.4	1229	1836	66.9
Among those who accessed or considered accessing mental health and wellness services						
Ability to access mental health and wellness services						
Not able to access services	144	776	18.6	332	1216	27.3
Sometimes able and sometimes not able to access services	312	776	40.2	581	1216	47.8
Always able to access services	320	776	41.2	303	1216	24.9
Among those who used substances Access to harm reduction services						
Accessed or considered accessing harm reduction services since the start of the COVID-19 pandemic	93	1016	9.2	152	1255	12.1

^a^Total represents total counts for the corresponding indicators excluding “don’t know”, “refused”, and “not stated” values, unless stated otherwise, which is why the total counts differ by indicator

### Access to care

Among participants with worsening mental health, 67% accessed or considered accessing mental health and wellness services. Among them, 75% reported difficulties, as they were “not able” or “sometimes able” to access these services since the start of the pandemic (Table 3). Among those who reported having the same or better mental health, 41.4% accessed or considered accessing mental health services, while 58.8% reported difficulties in accessing these services.

## Discussion

Results from three online surveys among PWUD, ACB, and FNIM people highlight the negative impact of the COVID-19 pandemic on social determinants of health, mental health, substance use, and access to care. PWUD, ACB, and FNIM participants reported worsening food insecurity, financial hardship, and feelings of safety since the start of the pandemic. Higher proportions of participants reported these experiences compared to general population surveys conducted by Statistics Canada. Almost half of survey participants reported that the COVID-19 pandemic had a major or moderate impact on their ability to pay bills. In comparison, as of February 2021, around one fifth of Canadians reported difficulty in paying expenses related to housing, food, transportation, or other expenses (Charnock et al., 2021). In 2021, 16% of Canadian families reported experiencing food insecurity, which increased to 18% in 2022 (Statistics Canada, 2023a). In contrast, half of participants from the impact of COVID-19 surveys reported experiencing some form of food insecurity since the start of the pandemic. In addition, 23% of survey participants reported feeling less safe where they lived during the COVID-19 pandemic. This proportion is greater than what was observed in a survey among the general Canadian population conducted in March and April 2020, where 8% reported being very or extremely concerned about the possibility of violence in their home (Statistics Canada, 2020).

Most survey participants who reported experiencing unfavourable social determinants of health before the pandemic reported that these experiences worsened during the pandemic, compared to those who reported more favourable social determinants of health before the pandemic. For example, 17.5% of those who felt very safe at home in the year before the pandemic reported feeling less safe since the start of the pandemic, compared to 31.6% of those who felt somewhat safe or did not feel safe before the pandemic, who felt even less safe during the pandemic. These findings reflect the historical pattern seen in previous epidemics, going back to the Black Death in the 1300s (Wade, 2020), the emergence of the HIV/AIDS epidemic in the 1980s (Crowley et al., 2021), and the 2003 global SARS outbreak (O’Sullivan & Phillips, 2019). People who were often already marginalized pre-pandemic—“the poor and minorities who face discrimination in ways that damage their health or limit their access to care”—have always borne the brunt of local and global pandemics. In turn, pandemics themselves affect societal inequality by reinforcing existing power structures and imbalances (Wade, 2020).

People in Canada faced heightened mental health challenges during the pandemic (PHAC, 2023a) and this was also reflected in these surveys. In addition to challenges related to deteriorating social determinants of health, survey participants also reported worsening mental health and increases in substance use. In particular, increases in substance use and in related behaviours such as using alone were higher among those with worsened mental health, compared to those with the same and better mental health. The reported increase in using substances alone is particularly alarming, as this behaviour is a risk factor for fatal overdose. Many of the survey participants with worsening mental health also reported an increase in the number of triggers for using substances. This is also alarming because alcohol and drug-related deaths reached new highs during the pandemic (Statistics Canada, 2023b), in the context of Canada already experiencing a surge in opioid-related deaths and other associated harms (PHAC, 2023c).

The demand for mental health services in Canada has significantly increased over time, accompanied by longer wait times for specialists (CBC, 2023). This high demand is coupled with increased difficulty in accessing services. A high proportion of survey participants were not always able to access mental health services when they considered using them. Importantly, the pandemic led to changes in the delivery of mental health and harm reduction services to key populations (Crowley et al., 2021; Matskiv et al., 2022). For example, some providers added telehealth for delivery of mental health services (PHAC, 2022). Innovations in the delivery of harm reduction services included virtual overdose monitoring services (Matskiv et al., 2022) and self-serve pick-up of harm reduction supplies at service window or curbside depots in some parts of Canada (PHAC, 2022). Further investigation is now needed to better understand how changes in service delivery models during the pandemic are affecting the ability of clients to get the care they seek. These can include impact evaluations to assess the reach of these interventions and whether changes in outcomes of interest (e.g. substance use–related harms) can be attributed to them (Harper & Nandi, 2017). Evaluations can be done by those involved in providing these services in collaboration with community members, researchers, and experts in policy impact assessments.

Findings from these surveys add to the growing evidence of health inequities faced by key populations in Canada during the COVID-19 pandemic and highlight the urgent need for action. While these surveys were cross-sectional in design with limited ability to assess causal links, the findings nonetheless contribute to our understanding of how the pandemic may have placed key populations, especially those with worsening mental health, at greater risk of poorer health. This is coupled with employment or income loss, increased food insecurity, and feeling less safe at home.

Upstream interventions and policies that promote health equity need to be prioritized to ensure they reach at-risk populations (PHAC, 2021). To reduce the health impact of STBBI in Canada by 2030 (PHAC, 2018) and to be better prepared for the next pandemic, incorporating an equity lens is essential. Applying an equity lens involves planning, implementing, and evaluating public health interventions through a perspective that prioritizes fairness, justice, and inclusivity. Practically, this should ensure an investment on health interventions based on the needs of equity-deserving populations that are in disadvantaged positions because of structural inequities leading to worsened social determinants of health. This can only be achieved through intersectoral collaboration across federal, provincial, territorial, and regional governments; Indigenous governments and communities; community-led organizations rooted within or working closely with populations disproportionately impacted by STBBI; and key stakeholders in relevant disciplines such as social sciences and economics (PHAC, 2021).

An example of a successful intersectoral collaboration includes the “COVID-19 Vulnerable Populations Task Group” (NL Health Services, 2023) that was formed by the provincial government of Newfoundland and Labrador in the early days of the pandemic. The group included representatives from all levels of government, health authorities, Indigenous governments, and community organizations. Among other tasks, the group developed COVID-19 guidance documents for key populations and ensured that all sectors remain informed of the impacts and gaps in services for marginalized communities. The group also established subcommittees focused on opioid dependency treatment, housing, and homelessness. Another example is the provincial Indigenous COVID-19 Collaboration table in Manitoba (Lavoie et al., 2023). The group consisted of representatives from Regional Health Authorities, FNIM organizations, and health system decision-makers from Manitoba, as well as Nunavut, to support a coordinated approach to pandemic planning, including how to roll out vaccination strategies in Indigenous communities where distrust of the health system is present (Lavoie et al., 2023). Although these groups were centered on the COVID-19 pandemic, they provide important learnings that could be applied to the STBBI field and beyond.

In terms of STBBI response, one of the guiding principles of the government of Canada’s STBBI action plan is health equity, where all people, regardless of sex, gender, race, income, and other characteristics, have equitable access to care (i.e. access based on the need for care) (PHAC, 2024). The continued implementation of the Truth and Reconciliation Commission’s Calls to Action (Truth and Reconciliation Commission of Canada, 2015) is another guiding principle. One of the priorities of the STBBI action plan is to support FNIM initiatives, leadership, and self-determination, in line with calls 18 to 24 pertaining to health care services, emphasizing the elimination of anti-Indigenous racism in the health care system and having a culturally competent care. Of note, the renewal of the STBBI action plan involved consultations with over 800 people across Canada in 2023, including members from key populations, health care professionals, members and representatives from community-based organizations, researchers, and activists. The action plan identifies federal priorities and actions grouped in four pillars (prevention, testing, initiation of care, and ongoing care) with nine federal departments responsible for their delivery. Each action is coupled with established indicators to monitor and measure progress from 2024 to 2030, with a commitment to work with key populations and community partners. This includes providing ongoing support using existing mechanisms, such as the Black Expert Working Group for National HIV Surveillance (PHAC, 2024).

One way to ensure the application of a health equity lens in prevention-based policies, programs, or interventions is by using the Health Equity Impact Assessment (HEIA) tool, specifically for STBBI (Canadian Public Health Association, 2014). This tool was developed by the Canadian Public Health Association in consultation and partnership with informants from key populations, public health professionals, clinicians, volunteers, and researchers. The tool is meant to support providers to ensure that their initiatives do not increase any existing health inequities. The HEIA includes five steps: scoping, thinking of unintended impacts, developing mitigation strategies, monitoring success, and disseminating results for improved equity and awareness. It highlights the importance of considering diverse populations, identifying factors impacting vulnerability, and proposing mitigation strategies to address negative impacts on key groups. The Chief Public Health Officer of Canada’s 2023 report on the State of Public Health in Canada highlights the HEIA tool as a health promotion tool for emergency management procedures (PHAC, 2023b). Indeed, a health promotion approach is emphasized as a strategy to strengthen both emergency management and population-level resilience. Supporting community involvement, providing sustainable funding, and incorporating Indigenous knowledge(s) are all essential components of this comprehensive approach (PHAC, 2023b).

### Limitations of the study

This work has some limitations. An anonymous online survey approach was used given the challenges of using probability-based sampling during the COVID-19 pandemic. The online nature of the surveys may have contributed to a selection bias, since those without access to a computer or internet would be less likely to participate. Also, because of convenience sampling, it is not possible to generalize the findings to all PWUD, ACB, and FNIM people in Canada. Together, this may have affected reported indicators. However, engaging with community stakeholders that promoted the surveys to help assure diverse representation across sub-populations may have mitigated some biases.

## Conclusion

For emergency response and STBBI-related prevention and care interventions to succeed for PWUD, ACB, and FNIM populations, it is crucial to work closely with communities. Through meaningful and sustainable relationships, it will be possible to develop equitable interventions that remain robust during a pandemic or other emergencies. When confronted with the next public health emergency, it will be these relationships that will allow for the timely mobilization of accessible services for key populations.

## Contributions to knowledge

What does this study add to existing knowledge?

- Findings from the three surveys add to the growing evidence of health inequities faced by key populations in Canada during the COVID-19 pandemic. The similarity of the surveys across three key populations allowed for a comprehensive assessment of the impact on social determinants of health, mental health, and substance use. Study findings highlight the pattern where equity-deserving populations bear the brunt of health crises.
- While this study sheds light on the challenges faced by key populations, there is a need for a more nuanced understanding, especially concerning the intersectionality of various identified factors affecting health outcomes. Additionally, exploring the long-term impacts of the pandemic on the health and well-being of these populations could provide insights for future public health planning and response strategies.

What are the key implications for public health interventions, practice, or policy?

- Current work on confronting STBBI epidemics and future pandemic preparedness plans should incorporate an equity lens. This includes considering social determinants of health, meaningfully collaborating with key populations when planning and implementing interventions, and securing contingency budgets for innovative service delivery models.
- Successful models of intersectoral collaboration, like the task groups from Newfoundland and Manitoba, highlight the importance of engaging representatives from various levels of government, health authorities, Indigenous governments, and community organizations, to ensure Public Health responses are comprehensive, equitable, and tailored to the unique needs of key populations.

## Data Availability

Not applicable.
